# Inadvertent Selection of a Pathogenic Fungus Highlights Areas of Concern in Human Clinical Practices

**DOI:** 10.3390/jof8020157

**Published:** 2022-02-05

**Authors:** Justin L. Eagan, Breanne N. Steffan, Sébastien C. Ortiz, Milton T. Drott, Gustavo H. Goldman, Christina M. Hull, Nancy P. Keller, Rafael W. Bastos

**Affiliations:** 1 Department of Medical Microbiology and Immunology, University of Wisconsin-Madison, Madison, WI 53706, USA; jeagan@wisc.edu (J.L.E.); bsteffan@wisc.edu (B.N.S.); mdrott@wisc.edu (M.T.D.); 2 Department of Biomolecular Chemistry, University of Wisconsin-Madison, Madison, WI 53706, USA; sortiz2@wisc.edu; 3 Faculdade de Ciências Farmacêuticas de Ribeirão Preto, Universidade de São Paulo, Ribeirão Preto 14040-903, Brazil; ggoldman@usp.br; 4 Department of Bacteriology, University of Wisconsin-Madison, Madison, WI 53706, USA; 5 Bioscience Center, Federal University of Rio Grande do Norte, Natal 59064-741, Brazil

**Keywords:** *Candida auris*, *Candida glabrata*, cross-tolerance, nosocomial infections, disinfectant cleaner, pathogen reservoir

## Abstract

In studying the development of tolerance to common hospital cleaners (Oxivir^®^ and CaviCide™) in clinical isolate stocks of the emerging, multidrug-resistant yeast pathogen *Candida auris*, we selected for a cleaner-tolerant subpopulation of a more common nosocomial pathogen, *Candida glabrata*. Through the purification of each species and subsequent competition and other analyses, we determined that *C. glabrata* is capable of readily dominating mixed populations of *C. auris* and *C. glabrata* when exposed to hospital cleaners. This result suggests that exposure to antimicrobial compounds can preferentially select for low-level, stress-tolerant fungal pathogens. These findings indicate that clinical disinfection practices could contribute to the selection of tolerant, pathogenic microbes that persist within healthcare settings.

## 1. Introduction

Opportunistic fungal pathogens are capable of rapid adaptation to environmental stressors in both clinical and non-clinical environments. A consequence of this ability is that conditions outside of human hosts can influence pathogen behavior(s) during infections. For example, azole-resistant *Aspergillus* spp. have been isolated from patients who had not received prior azole drug treatment [1,2,3,4], suggesting that the azole-resistant species had been selected in an environment that favored azole tolerance. One mechanism by which this can occur is through the use of azole-based agricultural pesticides; overuse of azoles on crops has also been implicated in cross-tolerance development in human pathogenic *Cryptococcus* spp. [5]. Cross-tolerance development is also a problem in hospital settings, where exposure to sub-lethal surface disinfectants, particularly quaternary ammonium compounds, can select for antibiotic-tolerant organisms and potentially lead to hospital-acquired infections [6,7]. The consequences of cross-tolerance have been particularly devastating for COVID-19 patients receiving immunosuppressive therapies who develop secondary infections with drug-resistant fungi. COVID-19 patients face substantially increased morbidity and mortality in the presence of nosocomial fungal disease [8,9,10,11,12].

Two of the most common causes of nosocomial fungal disease are *Candida glabrata* and *Candida auris. C. glabrata* is the most common non-albicans cause of candidiasis in the United States and northern Europe, with mounting evidence for increased prevalence over *Candida albicans* in some parts of the world [13]. Outbreaks of *C. auris* have occurred across the globe since its initial discovery in 2009 [14]. Most isolates of *C. auris* exhibit resistance to at least one class of antifungal drug, with approximately 90% of isolates showing resistance to fluconazole [15]. Multidrug resistance is rising in *C. auris* strains, with increasing reports of pan-resistant strains across many countries [15,16]. Importantly, environmental isolates of *C. auris* were recently shown to naturally exhibit multidrug resistance [17].

*Candida* species are particularly adept at persisting on abiotic surfaces [14,18,19]. To protect vulnerable patients from nosocomial infections, disinfection of clinical surfaces is key [7,20]; however, complete disinfection can be difficult to achieve, resulting in conditions favorable for the selection of stress-tolerant organisms. As stress tolerance can facilitate survival in previously unencountered environments, we hypothesize that repeated, low-level exposure to disinfecting agents can provide a selective environment in which *Candida* species develop tolerance to cleaners, resulting in concomitant altered tolerance to other conditions (e.g., antifungal drugs). To test this hypothesis, we evaluated two common hospital cleaners—namely, Oxivir^®^, approved for use against *C. auris* [21], and CaviCide™, which is used by hospitals in the local University of Wisconsin-Madison hospital system. Both agents are effective fungicides, with hydrogen peroxide as the primary active ingredient of Oxivir^®^ and quaternary ammonium salts as the primary active ingredients of CaviCide™. We passaged clinical *Candida* isolates through several generations using either cleaner as a source of stress and then evaluated the resulting populations for the consequences of adaptation.

We discovered that *C. auris* and *C. glabrata* respond differently to hospital cleaner exposure. Our passaging experiments unexpectedly selected for a trace abundance of contaminating *C. glabrata* in our original *C. auris* stocks. Through purification and characterization of the strains, we discovered that *C. glabrata* was intrinsically more tolerant to cleaners than *C. auris* and showed different tolerances to antifungal agents. Finally, we determined in direct competition experiments that *C. glabrata* outcompetes *C. auris* in the presence of hospital cleaners. These findings demonstrate how abiotic stress can influence mixed-community dynamics and highlight the importance of considering mixed *Candida* communities in clinical environments and nosocomial disease.

## 2. Materials and Methods

### 2.1. Strains and Culturing

Both *Candida auris* strains B11804 (C54007) and B11785 (C48321) originate from a panel of clinical isolates from the Centers for Disease Control and Prevention (both Clade IV). Original CDC glycerol stocks were cultured and used to generate secondary stocks from which we created our laboratory working stocks. We grew these strains statically in 1 mL cultures of YPD media within 24-well plates at 30 °C for 48 h. For populations passaged in hospital cleaners, they were grown in YPD amended with MIC value of the respective cleaner for the same duration.

### 2.2. Passaging Experiments

For each *C. auris* strain, working glycerol stocks stored at −80 °C were streaked out on solid YPD plates and used to inoculate 1 mL YPD cultures for 48-h incubation at 30 °C. Cultures were collected in 1.5 mL tubes, washed once with 0.85% NaCl and resuspended in 0.85% NaCl. All centrifugation was at 10,000 rpm for 10 min. 100 µL of cell suspensions were added to 96-well plates for quantifying optical density at 600 nm (OD_600_) with a BioTek^®^ (Winooski, Vermont) EPOCH 2 microplate reader. The OD_600_ values were used to measure the average yeast/mL based on a standard curve we established in the beginning. This standard curve involved reading the OD_600_ values and correlating this with the number of cells enumerated with a hemocytometer. We calculated appropriate dilutions to inoculate 25,000 cells in 1 mL of unamended YPD or YPD amended with a hospital cleaner. We inoculated each strain into YPD alone, YPD + Oxivir^®,^ and YPD + CaviCide™, in triplicate. Our first cultures were inoculated at each strain’s 0.25× MIC value for one 48-h incubation period, followed by another passage at 0.5 × MIC values before beginning the first actual passage at the MIC values of each cleaner. This procedure was conducted with the intention of allowing the strains to adapt to the cleaners before applying fungicidal concentrations. Our methodology for determining MIC values is described below, which includes the evaluation of our original stocks.

### 2.3. Minimum Inhibitory Concentration Assays

The protocol detailed for microdilution antibiotic susceptibility testing by the Clinical and Laboratory Standards Institute (M27 Ed4) [22] was utilized for our MIC assays with 2500 cells/mL of YPD amended with hospital cleaner at 30 °C or RPMI-MOPS (pH 7) amended with antifungals at 37 °C. MIC values for fluconazole (fungistatic) represent MIC_50_, while all other MIC values reported are MIC_100_. Each population’s MIC value was measured twice per antifungal agent for each assay, with final MIC values for the population represented as the mode value. Raw MIC values for all populations are provided in the Supplementary Materials (Table S1).

### 2.4. Flow Cytometry

We followed the protocol detailed by Almeida et al. [23], with some modifications. Briefly, cells were fixed overnight in 70% ethanol at 4 °C, followed by resuspension in 850 µL 50 mM sodium citrate (pH 7.5). Cell suspensions were then gently sonicated and treated with RNase A, before staining overnight with SYBR Green at 4 °C. Triton X-100 was added to each sample (0.25% *v*/*v*) and then ran at 30,000 cells/sample on a FITC channel on low flow rate with a linear scale. Data were analyzed using FlowJo (v10.7.1).

### 2.5. Whole-Genome Sequencing

Each selected population was grown in 10 separate 1 mL YPD or YPD amended with appropriate MIC value hospital cleaner at 30 °C for 48 h. Cultures were pooled together and washed once with 0.85% NaCl, before extracting gDNA with phenol–chloroform. Samples were treated with RNase and analyzed with a high-affinity DNA Qubit kit before submitting to Novogene (Beijing, China) for Illumina sequencing (NovaSeq 6000 PE150).

### 2.6. Purification and Verification of C. auris and C. glabrata Isolates

Populations 1.2 YPD and 1.1 OX from the fifth passages (passaging experiment 1) were plated onto YNB media with 10% NaCl (*w*/*v*) and either glucose or mannitol as the sole carbon source, based on work from Welsh et al. (2017) [24]. Three colonies from the 1.2 YPD population were selected from a YNB mannitol plate, and three colonies from the 1.1 OX population were selected from a YNB glucose plate. These six isolated colonies were streaked out on YPD to obtain single colonies. Single colonies were selected of each and inoculated into 1 mL YPD media to grow for 48 h at 30 °C. Cultures were collected, washed once with 0.85% NaCl, and resuspended in 1 mL 0.85% NaCl, to create glycerol stocks. The remaining cells were used to extract gDNA for PCR analysis. Species-specific ITS-region primers were designed based on our sequence data of the populations. *C. auris*-specific primers were as follows: forward: 5′-acctgcggaaggatcattattgaagc-3′ and reverse: 5′-ttaagttcagcgggtagtcctacc-3′. *C. glabrata*-specific primers were as follows: forward: 5′-ttaagttcagcgggtaaccctacc-3′ and reverse: 5′-taagtgcgcggttggtgg-3′. CHROMAgar™ Candida (BD BBL™) differential plates were also used for diagnostic verification by streaking and incubating for 48 h at 30 °C.

### 2.7. Defined Mixture Passaging Experiment and Relative Proportion Determination

One verified isolate of both *C. auris* and *C. glabrata* was mixed at a 50:50 ratio, based on average yeasts/mL from our standard curve. This mixture was inoculated in triplicate into 1 mL cultures of unamended YPD and YPD, amended with 0.6% CaviCide™ at 25,000 cells per ml. Each passage was incubated at 30 °C in a static 24-well plate for 48 h. Similar to our passaging procedure, each passage was collected and washed with 0.85% NaCl before inoculating 25,000 cells into the next passage. For every passage, as well as the initial mixture, each population was plated onto 10 YNB + glucose plates and 10 YNB + mannitol plates. These plates were incubated at 30 °C for 48 h before enumerating CFUs. To compare the relative ratio of *C. auris* between unamended YPD-passaged populations and 0.6% CaviCide™ amended YPD-passaged populations, we performed multiple unpaired student *t* tests at each passage using GraphPad Prism (San Diego, CA, USA).

## 3. Results

### 3.1. Serial Exposure of C. auris Clinical Isolates to Hospital Cleaners Selected for C. glabrata Outgrowth

**Figure 1:**
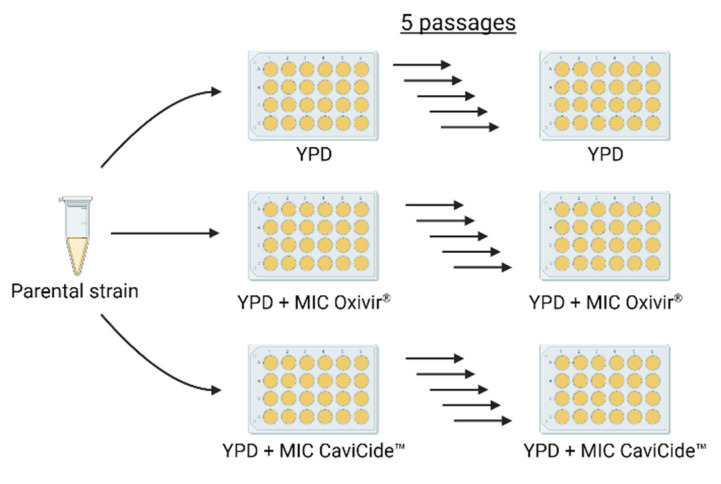
Schematic of serial passaging experimental design. Created with BioRender.com.

To determine the effects of hospital cleaners on cross-tolerance development, we carried out two independent passaging experiments. In each experiment, we grew two clinical isolates of *C. auris* (B11804 and B11785) in serial liquid cultures. Three independent cultures for each isolate were grown in either YPD medium alone or YPD medium containing minimum inhibitory concentrations (MICs) of either Oxivir^®^ or CaviCide™ cleaning agents (Figure 1).

**Figure 2:**
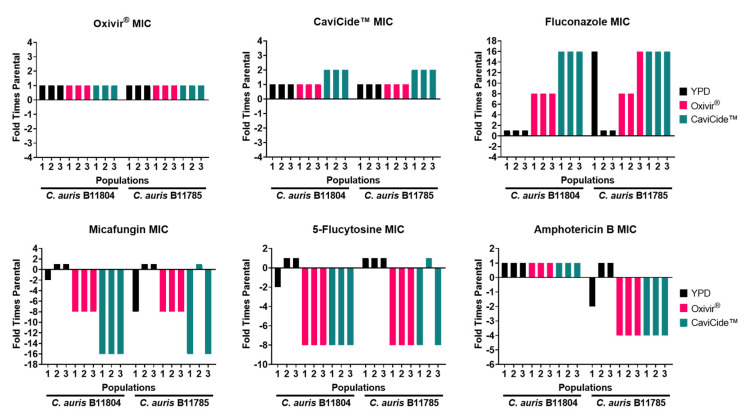
MIC assays of passaging experiment 1 populations. Each bar represents an individual population passaged in the media indicated by the legend.

After five serial passages of two days each, the populations were collected and compared to our unpassaged, parental populations. We first tested the ability of our passaged populations to grow in the presence of six different antifungal agents, including Oxivir^®^, CaviCide™, fluconazole, micafungin, 5-flucytosine, and amphotericin B. From our first passaging experiment (Figure 2), we discovered that the growth of B11804 cultures serially grown in the presence of unamended YPD (black bars) was not different from the growth of unpassaged parental strain (a twofold change in MICs was not considered significant). In contrast, all three B11804 populations grown serially in the presence of Oxivir^®^ (fuchsia bars) showed an eightfold increase in tolerance to fluconazole and an eightfold increase in susceptibility to both micafungin and 5-flucytosine. Similarly, B11804 populations grown in the presence of CaviCide™ (teal bars) developed a 16-fold greater tolerance to fluconazole and 16- and 8-fold decreases in tolerance to micafungin and 5-flucytosine, respectively. Similar shifts in antifungal susceptibility profiles occurred in B11785 populations, correlating primarily with cleaner exposure. Overall, we found that cleaner-passaged populations developed higher tolerances to fluconazole and lower tolerances to micafungin, 5-flucytosine, and amphotericin B.

**Figure 3:**
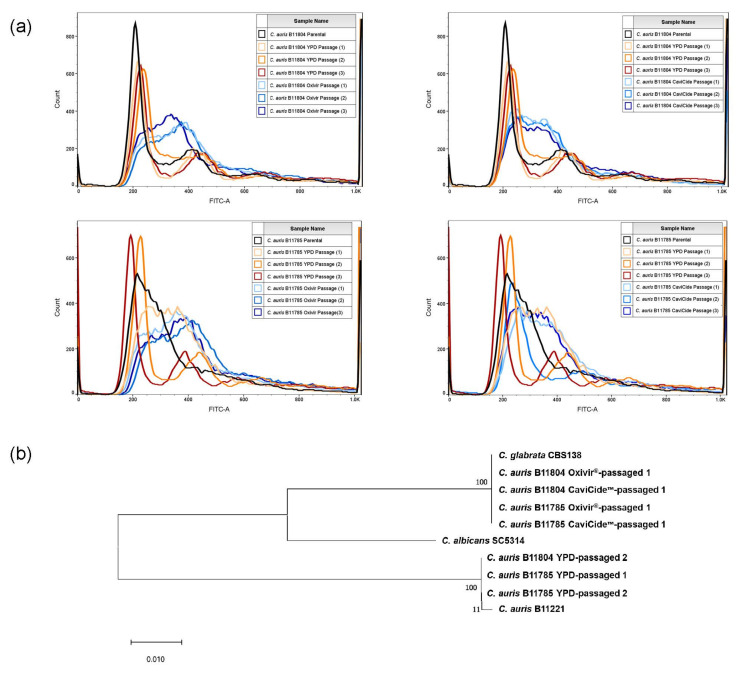
Cleaner-passaged populations are dominated by *C. glabrata*: (**a**) flow cytometry of populations from passaging experiment 1; (**b**) phylogeny of sequencing reads from cleaner-passaged populations and their relatedness to *C. glabrata* and *C. auris* type strain sequences, with *C. albicans* SC5314 as an outgroup.

Changes in ploidy and other chromosomal rearrangements have been associated with the development of antifungal drug resistance in other species [25,26]; thus, we performed flow cytometry analyses. Our cleaner-passaged populations exhibited increased overall DNA content, suggesting that a subpopulation may have undergone some degree of chromosomal duplication (Figure 3a). We hypothesized that aneuploid development may have occurred in our cleaner-passaged populations, resulting in the observed antifungal drug susceptibility alterations. To test this hypothesis, we selected seven populations for Illumina whole-genome sequencing with the intention of performing copy number variation analysis. To our surprise, we discovered the cleaner-passaged populations’ reads matched *C. glabrata* rather than *C. auris* (Figure 3b). These findings suggested that the stocks of *C. auris* from which the passaging experiment was inoculated were contaminated with an otherwise-undiscernible level of *C. glabrata*, which we identified as strain BG2 through molecular typing (Table S2).

A second, independent passaging experiment with the same *C. auris* stocks resulted in populations exhibiting similar antifungal MIC profiles, where exposure to cleaners altered the susceptibility profiles of the populations, compared to parental strains (Figure S1). Further investigation into our populations indicated that the cleaner-passaged, *C. glabrata*-dominated populations demonstrated increased tolerance to the cell-wall stressors Congo Red and calcofluor white (Figure S2), exhibited faster growth rates (Figure S3), and overall contained more elongated cell shapes (Figure S4) than the starting cultures.

To determine whether the population properties were transient, we passaged the cleaner-passaged experiment 1 populations in YPD without cleaners and found the antifungal profiles to remain stable even after 30 passages (Figure S5), suggesting that *C. glabrata* was firmly established as the predominant, stable microbe in the cultures, even in the absence of further selection. In fact, we discovered that (1) *C. glabrata* dominated cleaner-passaged populations, (2) dominance by *C. glabrata* under cleaner selection occurred with both test strains of *C. auris*, and (3) *C. glabrata* dominance was a repeatable phenomenon, occurring in independent experiments carried out by different researchers using identical methodologies.

### 3.2. C. glabrata Tolerated Higher Concentrations of Hospital Cleaner than C. auris

To understand the source(s) and properties of the *Candida* species in our experimental cultures, we used *C. auris* selective media [24] to isolate pure *C. auris* and pure *C. glabrata* from two of the previously sequenced populations from passaging experiment 1. Our pure *C. auris* isolates originated from a YPD-passaged population, while our pure *C. glabrata* isolates originated from an Oxivir^®^-passaged population. Species-level identification of the isolates was confirmed with CHROMagar™ Candida plates as well as species-specific ITS region PCR primers (Figure S6).

**Figure 4:**
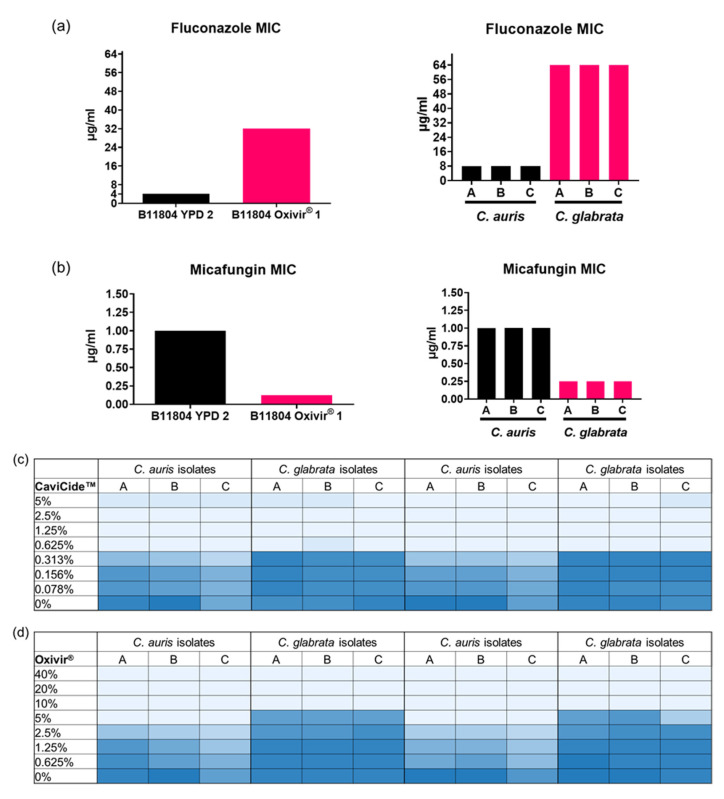
*Candida glabrata* correlates with original cleaner-passaged populations’ MIC data and tolerates higher concentrations of both cleaners: (**a**) fluconazole MIC assay with original populations (left) compared to purified *C. auris* and *C. glabrata* isolates (right); (**b**) Micafungin MIC assays comparing original populations to purified isolates; (**c**) CaviCide™ MIC assay of purified isolates with each cell representing the plate well, and the darker the cell, the higher the OD_600_ reading; (**d**) Oxivir^®^ MIC assay setup just as (**c**).

We found the results of our original MIC assays with increased tolerance to fluconazole and decreased tolerance to micafungin in our cleaner-passaged populations correlated with our *C. glabrata* purified isolates (Figure 4a,b). Next, we hypothesized that the domination of *C. glabrata* in the cleaner-passaged populations was the result of an intrinsic property of the *C. glabrata* isolates to tolerate cleaner treatment better than the *C. auris* isolates. Thus, we performed CaviCide™ and Oxivir^®^ MIC assays to verify the MIC values of our pure *C. auris* and *C. glabrata* isolates. While the CaviCide™ MIC values of our pure isolates matched our previous values of 0.625%, *C. glabrata* isolates grew markedly more robustly at higher concentrations of CaviCide™, compared with *C. auris* (Figure 4c). Importantly, we determined that the pure *C. auris* isolate had a MIC_100_ in 5% Oxivir^®^ (matching the original stock culture MIC_100_), but the MIC_100_ value for the pure *C. glabrata* isolates was higher (10% Oxivir^®^) (Figure 4d). These data suggest that the higher tolerance of *C. glabrata* to our cleaners in the original culture may explain the dominance over *C. auris* strains in the cleaner-passaged populations.

### 3.3. C. auris Was Quickly Outgrown in Cleaner-Passaged Mixed Populations

**Figure 5:**
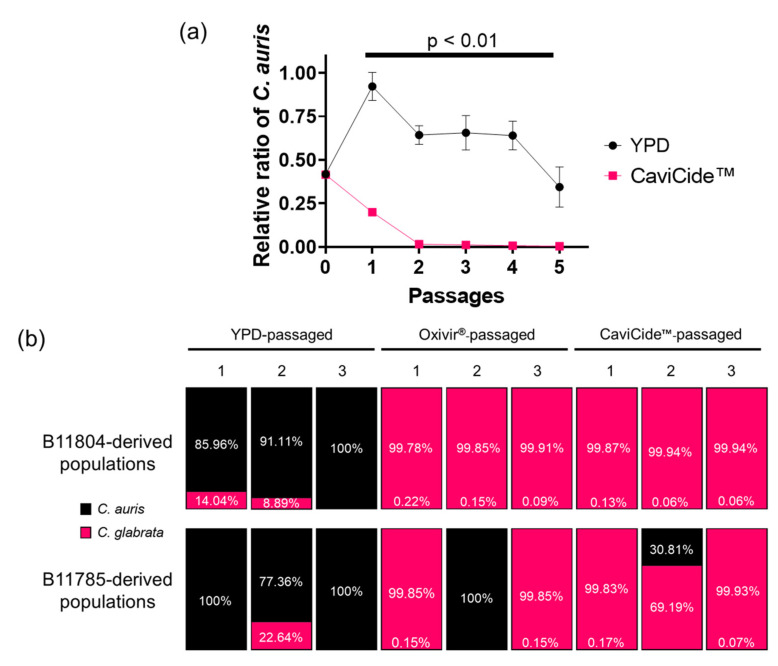
*C. glabrata* consistently outgrows *C. auris* upon cleaner exposure: (**a**) relative proportion of *C. auris* at each passage of the defined-mixture passaging experiment. Student’s *t*-test was used to determine the significance of relative ratio difference at each passage between cleaner- and YPD-passaged populations; (**b**) retrospective analysis of populations from passaging experiment 1 with relative proportions of each species.

To test the hypothesis that *C. glabrata* can outcompete *C. auris* to become the dominant species in a mixed population upon hospital cleaner exposure, we established a defined ratio of *C. auris* and *C. glabrata* and monitored relative ratios of the strains throughout selective passaging. We passaged the defined ratio mixture in triplicate through YPD and YPD with CaviCide™ at the MIC value (0.625%) for five passages (Figure S7). We chose CaviCide™ as our cleaner in this experiment because our *C. glabrata* isolate was purified from an Oxivir^®^-passaged population, and the MIC value is the same between both the *C. auris* and *C. glabrata* isolates. Each passage was plated onto minimal media and *C. auris* selective media, to enumerate total colonies, compared with *C. auris* colonies. We observed a rapid decrease in *C. auris* abundance in the cleaner-passaged populations (Figure 5a). In a retrospective analysis of our previous passaging experiments, we observed a similar trend in which cleaner-passaged populations were dominated by *C. glabrata* overall (Figure 5b). These data correlated with our sequenced populations, as well as the defined mixture experiment, suggesting that *C. glabrata* is indeed capable of dominating mixed populations of *C. auris* and *C. glabrata* at varying ratios when passaged in hospital cleaners.

## 4. Discussion

Here, we present our findings that two of the most common causative agents of hospital-acquired candidiasis, *C. auris* and *C. glabrata*, show different responses to environmental stress caused by exposure to hospital cleaners. Passaging of clinical isolates of *C. auris* (contaminated with *C. glabrata*) in Oxivir^®^ and CaviCide™ resulted in the unexpected selection of a cleaner-tolerant *C. glabrata* strain. This finding drove our investigation away from our original intention of *C. auris* cross-tolerance development and toward mixed-population dynamics in the presence of abiotic stress. Through purification and characterization of these strains, we determined that the *C. glabrata* isolate was intrinsically more tolerant to cleaners than *C. auris* and outcompeted *C. auris* in direct competition experiments.

We determined that the contaminating strain was *C. glabrata* BG2 in both of our *C. auris* glycerol stocks (Table S2). BG2 is a clinical isolate from a patient whose infection did not respond to fluconazole treatment [27], likely explaining the dramatic decrease in susceptibility to fluconazole of our cleaner-passaged populations (Figure 2 and Figure S1). Attempts to identify the point and source of contamination were inconclusive; however, we hypothesized that our stocks of *C. auris* were contaminated prior to initiation of our experiments because *C. glabrata* was recovered from cleaner-passaged populations in two independent passaging experiments. Our retrospective analyses support this, as we detected *C. glabrata* present in YPD-passaged populations, in both backgrounds, from both passaging experiments (Figure 5b and Figure S8).

The presence of *C. glabrata* in our passaged populations explains the variability we saw in our MIC assays (Figure 2 and Figure S1), where relative proportions of the two species varied within each population (Figure 5b and Figure S8). The relative ratio of each species also contributed to population-level phenotype discrepancies. An example of this can be seen comparing passaging experiment 1 B11785 CaviCide™-passaged population 1 versus population 2, where the former is almost completely *C. glabrata*, and the latter still contains approximately 30% *C. auris* (Figure 5b). As a result, we observed increased tolerance to Congo Red and calcofluor white in the first population, whereas the population containing more *C. auris* exhibited similar susceptibility to the parental *C. auris*-dominant strain (Figure S2). This provides an example of how abiotic stress exposure on a mixed population can rapidly select for strains tolerant to the stressor, thereby altering the overall population’s response to antifungal agents.

Unintentional selection of microorganisms as a byproduct of human practices is a well-documented occurrence, particularly in the context of antimicrobial compounds employed in clinical and agricultural settings that have been linked to the emergence of cross-tolerant strains [1,5]. Similarly, the application of hospital cleaners has been implicated in selecting for tolerance in a clinical setting, which may have implications for resistance emergence [28]. We intend this study to act as a cautionary tale on the ease with which pathogenic fungi may be unintentionally selected for by human interventions, particularly in a clinical context. Considering hospitals as potential reservoirs for pathogens [20,29,30], it is critical to frame our discussions on pathogen resistance emergence around the ecology of healthcare facilities and the selective forces we may be applying to these microbial populations. As researchers continue the important work of studying *C. auris* and other emergent fungal pathogens, it is imperative that the wide-ranging potential consequences of stringent selection be considered when interpreting experimental outcomes.

## Data Availability

Not applicable.

## Supplementary Materials

The following are available online at www.mdpi.com/article/10.3390/jof8020157/s1, Table S1: Raw MIC values of each population from passaging experiments, Table S2: PubMLST analysis of sequenced populations that matched *C. glabrata*, Figure S1: MIC assays of passaging experiment 2 populations, Figure S2: Cell-wall stress tolerance assays, Figure S3: Growth curve assays, Figure S4: Cell shape analyses, Figure S5: MIC assays of cleaner-passaged populations (experiment 1) after additional passaging in YPD alone, Figure S6: Verification of *C. auris* and *C. glabrata* isolates, Figure S7: Passaging experimental setup with defined ratios of *C. auris* and *C. glabrata*, Figure S8: Retrospective analysis of populations from passaging experiment 2.
