# Supplementary material for: Inadvertent Selection of a Pathogenic Fungus Highlights Areas of Concern in Human Clinical Practices
Source: J Fungi (Basel). 2022 Feb 5;8(2):157. doi: 10.3390/jof8020157 (PMC8878328; doi:10.3390/jof8020157)
Supplement: Supplementary file 1 [file jof-08-00157-s001.zip › jof-1543537-Supplementary.pdf]

		Oxivir® (%)	CaviCide™ (%)	Fluconazole (µg/ml)	Micafungin (µg/ml)	5-Flucytosine (µg/ml)	Amphotericin B (µg/ml)
Passaging Experiment 1	B11804 parental	10	0.625	4	1	0.5	1
	B11804 YPD 1	10	0.625	4	0.5	0.25	1
	B11804 YPD 2	10	0.625	4	1	0.5	1
	B11804 YPD 3	10	0.625	4	1	0.5	1
	B11804 OX 1	10	0.625	32	0.125	0.0625	1
	B11804 OX 2	10	0.625	32	0.125	0.0625	1
	B11804 OX 3	10	0.625	32	0.125	0.0625	1
	B11804 CAV 1	10	1.25	64	0.0625	0.0625	1
	B11804 CAV 2	10	1.25	64	0.0625	0.0625	1
	B11804 CAV 3	10	1.25	64	0.0625	0.0625	1
	B11785 parental	10	0.625	4	1	0.5	4
	B11785 YPD 1	10	0.625	64	0.125	0.5	2
	B11785 YPD 2	10	0.625	4	1	0.5	4
	B11785 YPD 3	10	0.625	4	1	0.5	4
	B11785 OX 1	10	0.625	32	0.125	0.0625	1
	B11785 OX 2	10	0.625	32	0.125	0.0625	1
	B11785 OX 3	10	0.625	64	0.125	0.0625	1
	B11785 CAV 1	10	1.25	64	0.0625	0.0625	1
	B11785 CAV 2	10	1.25	64	1	0.5	1
	B11785 CAV 3	10	1.25	64	0.0625	0.0625	1
Passaging Experiment 2	B11804 parental	5	1.25	8	0.25	2	2
	B11804 YPD 1	10	1.25	4	0.25	2	2
	B11804 YPD 2	10	1.25	4	0.25	2	2
	B11804 YPD 3	10	1.25	4	0.25	2	2
	B11804 OX 1	10	0.625	64	0.0625	0.125	2
	B11804 OX 2	10	0.625	64	0.0625	0.125	2
	B11804 OX 3	10	0.625	64	0.0625	0.125	2
	B11804 CAV 1	10	2.5	8	0.25	1	2
	B11804 CAV 2	10	2.5	8	0.25	1	2
	B11804 CAV 3	10	2.5	64	0.5	1	2
	B11785 parental	10	1.25	4	0.25	2	4
	B11785 YPD 1	5	1.25	4	0.25	2	4
	B11785 YPD 2	5	1.25	4	0.25	2	4
	B11785 YPD 3	10	1.25	4	0.25	1	4
	B11785 OX 1	10	0.625	4	0.25	2	4
	B11785 OX 2	10	0.625	4	0.25	2	4
	B11785 OX 3	10	0.625	4	0.25	2	4
	B11785 CAV 1	5	2.5	8	0.25	2	4
	B11785 CAV 2	10	5	4	0.25	2	4
	B11785 CAV 3	10	5	32	0.25	2	4

Table S1. Raw MIC values (mode of technical replicates) for each population from both passaging experiments.

Strain	Allele for each locus						Sequence Type
	FKS	LEU2	NMT1	TRP1	UGP1	URA3	
C. glabrata CBS138	8	5	3	5	1	1	15
C. glabrata BG2	5	7	8	7	3	6	3
B11804 Oxivir®-passaged 1	5	7	8	7	3	6	3
B11804 CaviCide™-passaged 1	5	7	8	7	3	6	3
B11785 Oxivir®-passaged 1	5	7	8	7	3	6	3
B11785 CaviCide™-passaged 1	5	7	8	7	3	6	3

Table S2. PubMLST analysis of sequenced populations that matched *C. glabrata* from passaging experiment 1.

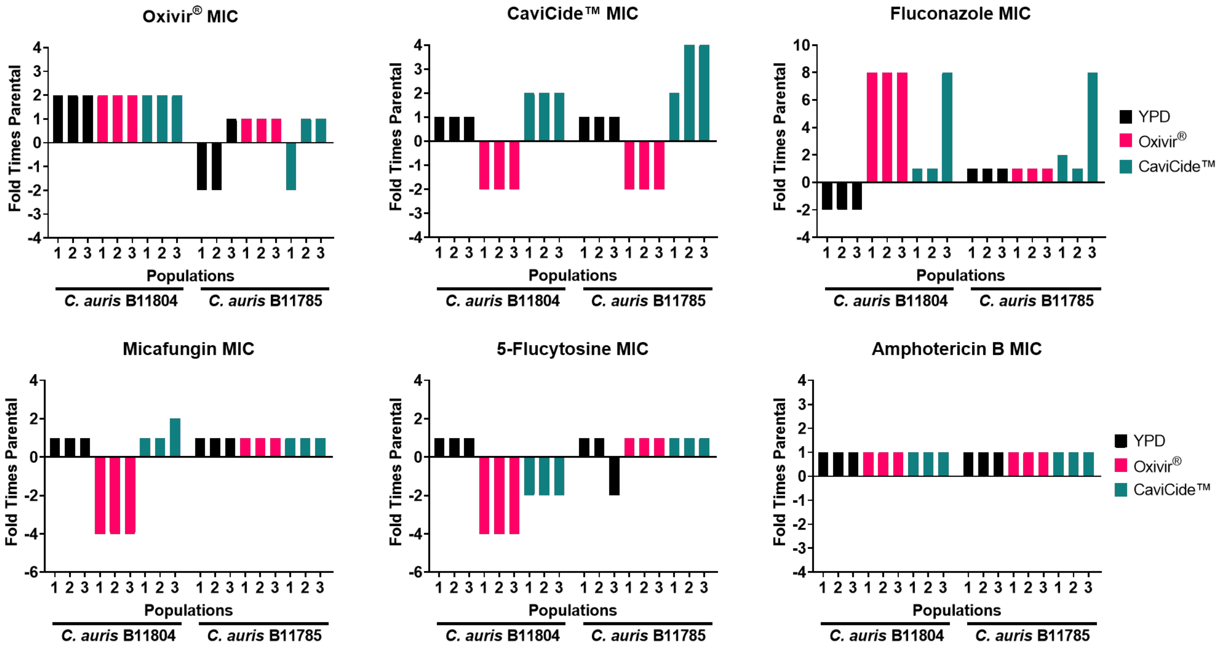

Figure S1. MIC assays of passaging experiment 2 populations. Each bar represents an individual population passaged in the media indicated by the legend.

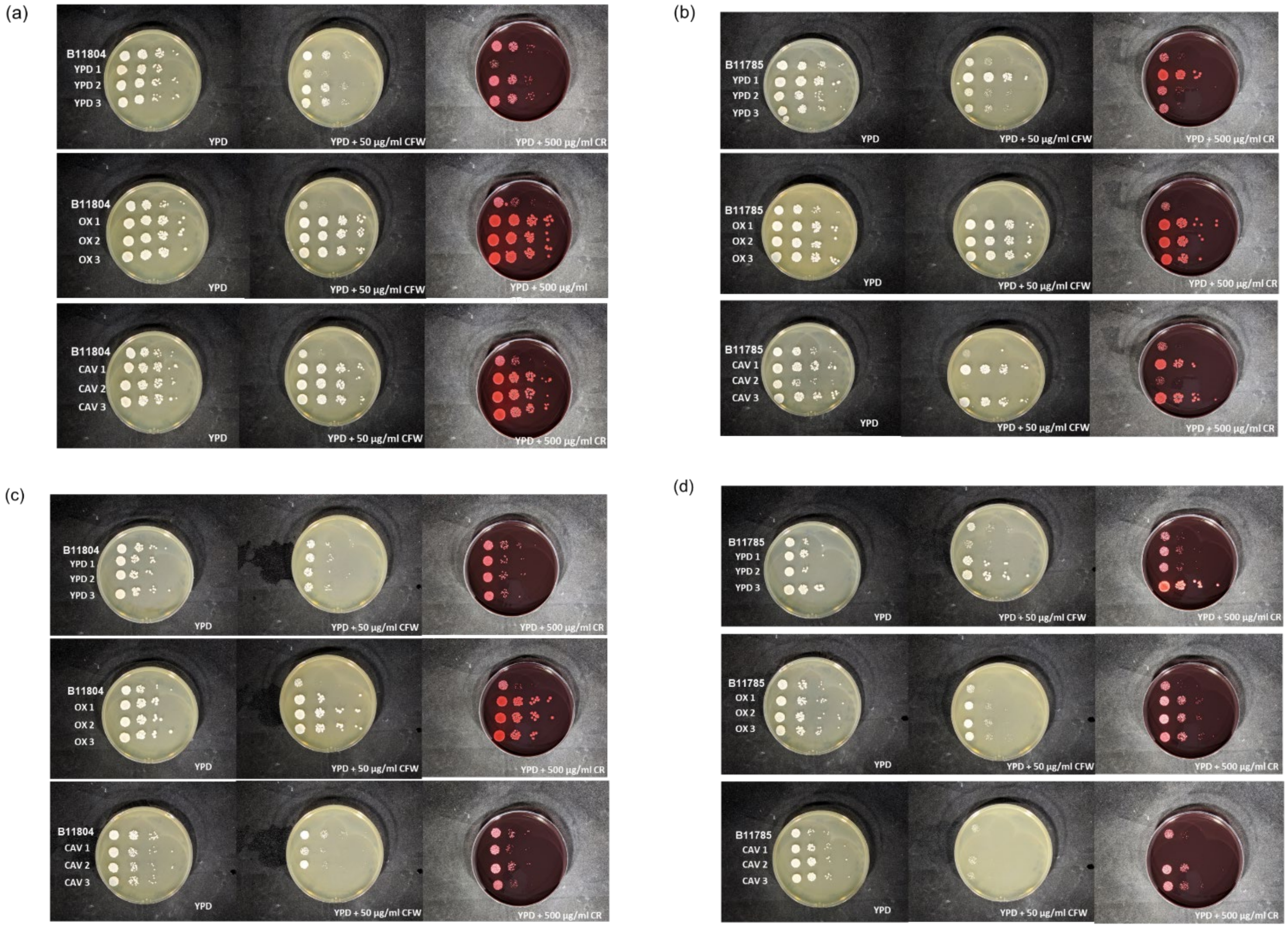

Figure S2. Cell wall stress tolerance assays with calcofluor white (CFW) and Congo Red (CR). (a) B11804-derived populations from passaging experiment 1. (b) B11785-derived populations from passaging experiment 1. (c) B11804-derived populations from passaging experiment 2. (d) B11785-derived populations from passaging experiment 2.

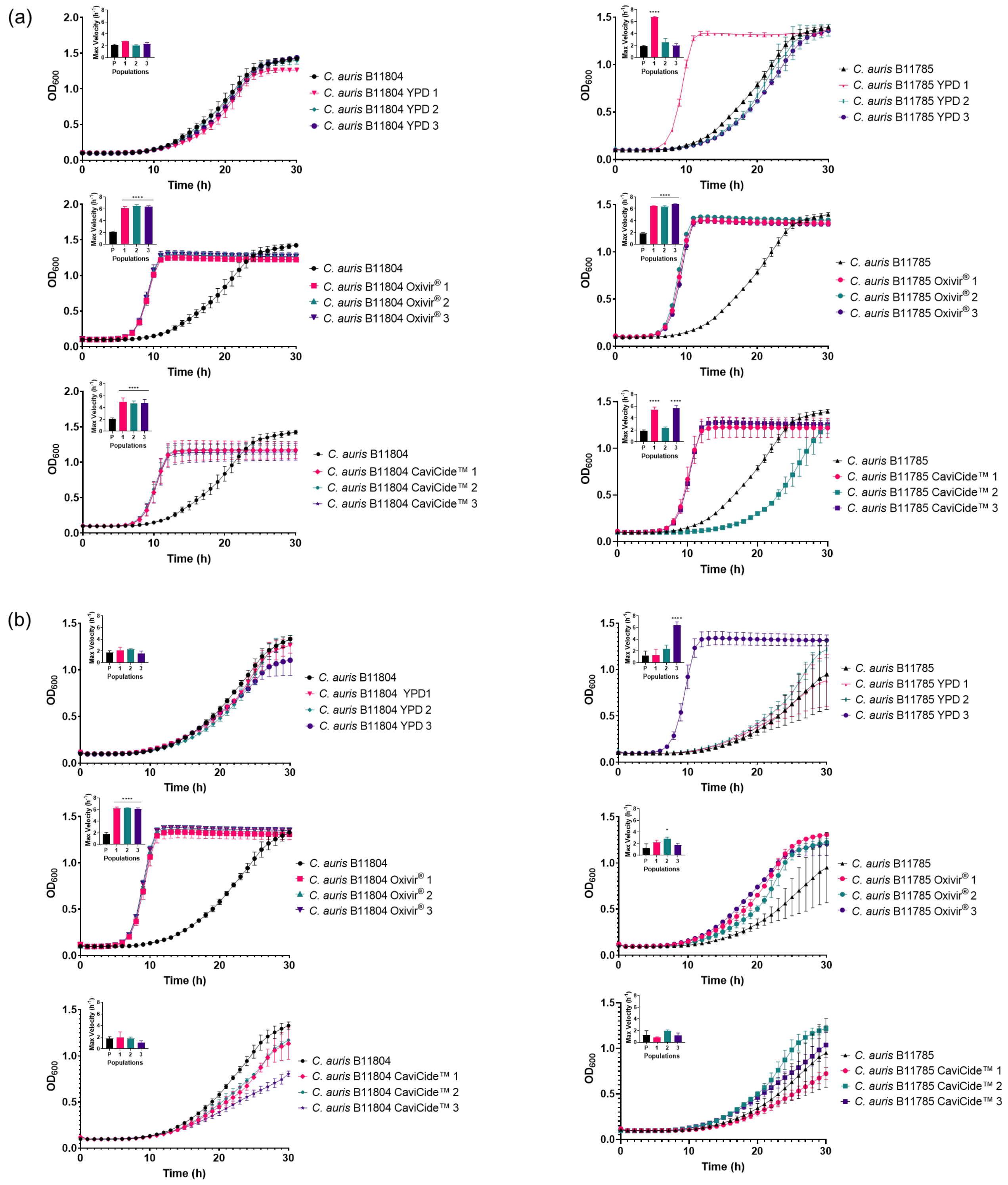

Figure S3. Growth curve assays with max velocity data inset. (a) Populations from passaging experiment 1. (b) Populations from passaging experiment 2.

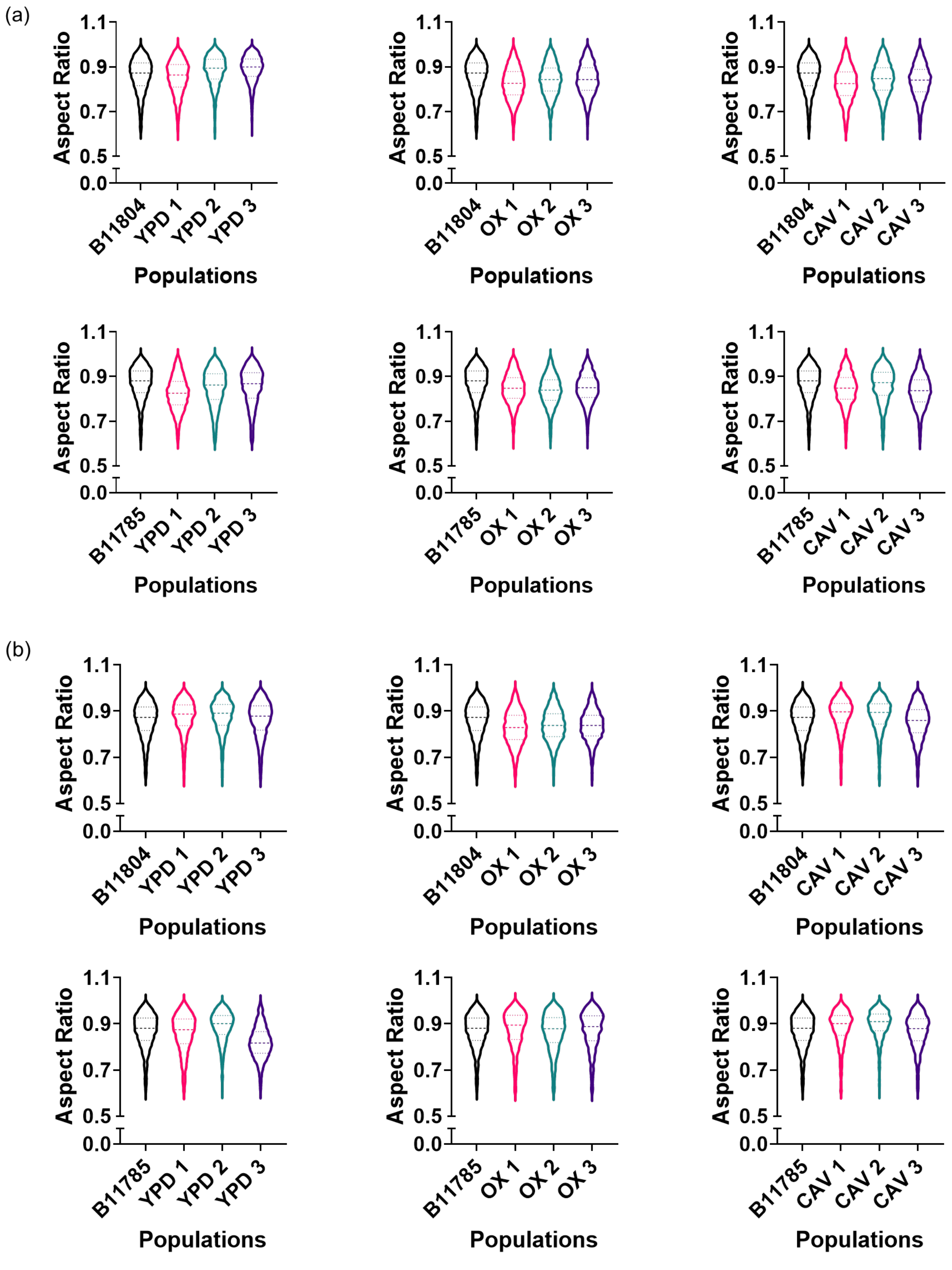

Figure S4. Cell shape analyses. (a) Populations from passaging experiment 1. (b) Populations from passaging experiment 2.

(a)

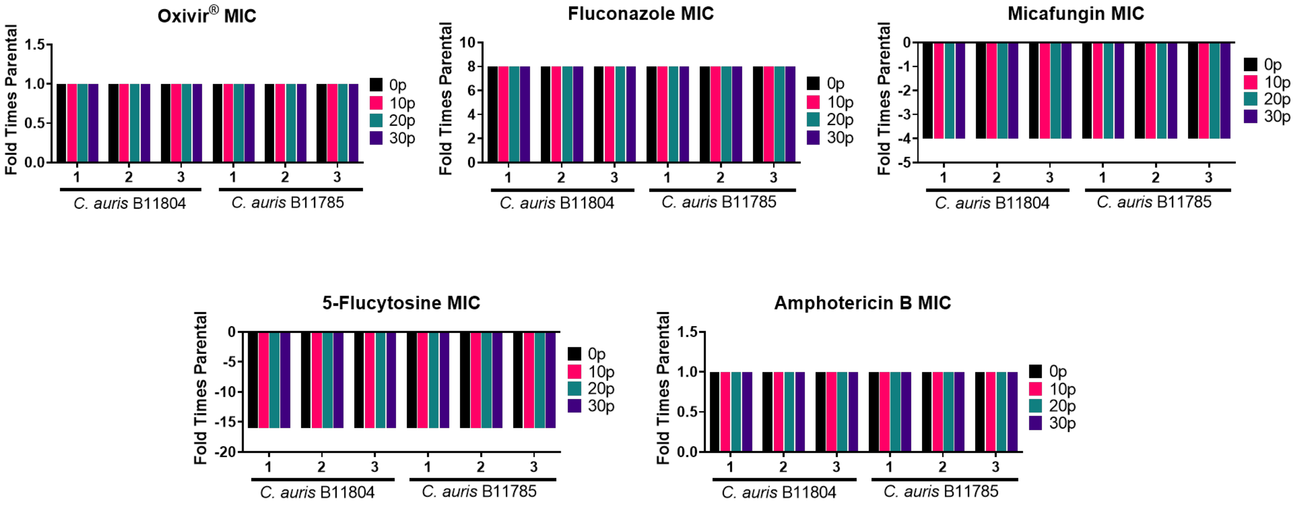

(b)

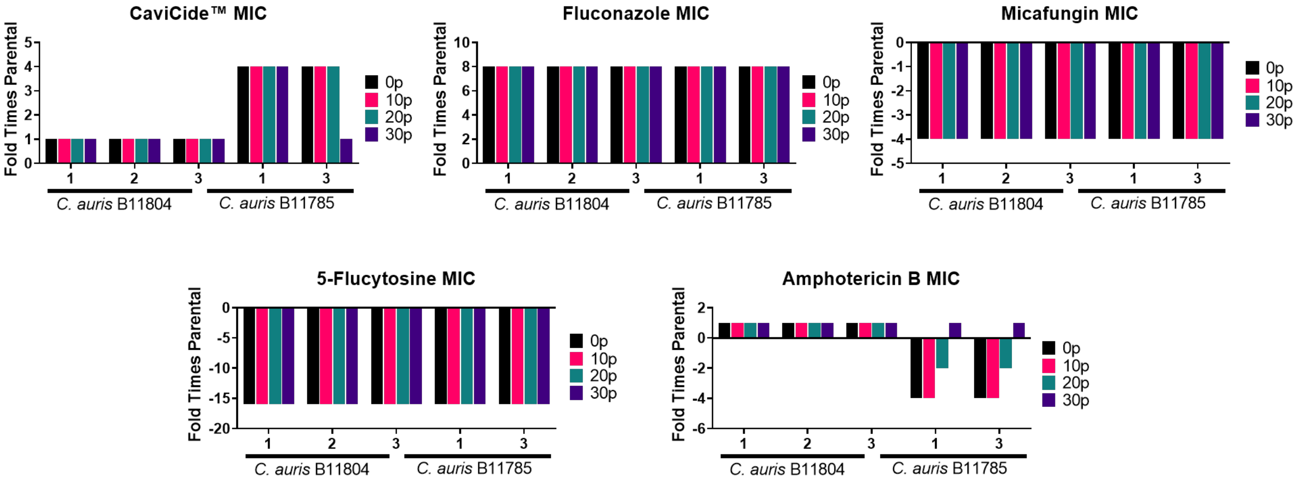

Figure S5. MIC assays of cleaner-passaged populations (experiment 1) after additional passaging in YPD alone. (a) Oxivir®-passaged populations. (b) CaviCide™-passaged populations.

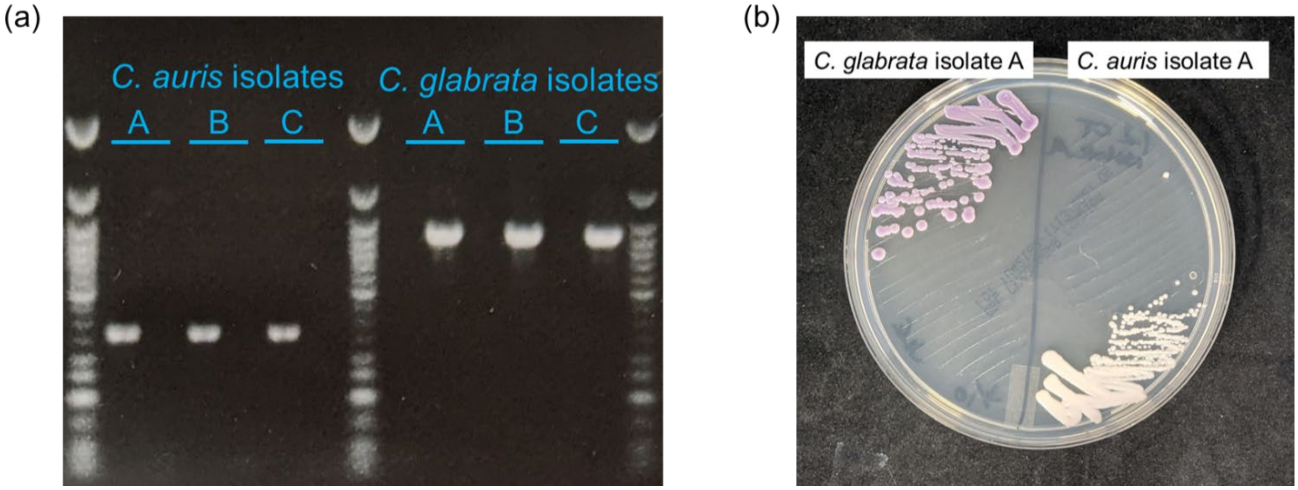

Figure S6. Verification of *C. auris* and *C. glabrata* isolates. (a) PCR-verification of isolates with ITS-specific primers. All isolates were tested with *C. auris*-specific primers (left) and *C. glabrata*-specific primers (right). (b) CHROMagar™ Candida phenotypes of isolates.

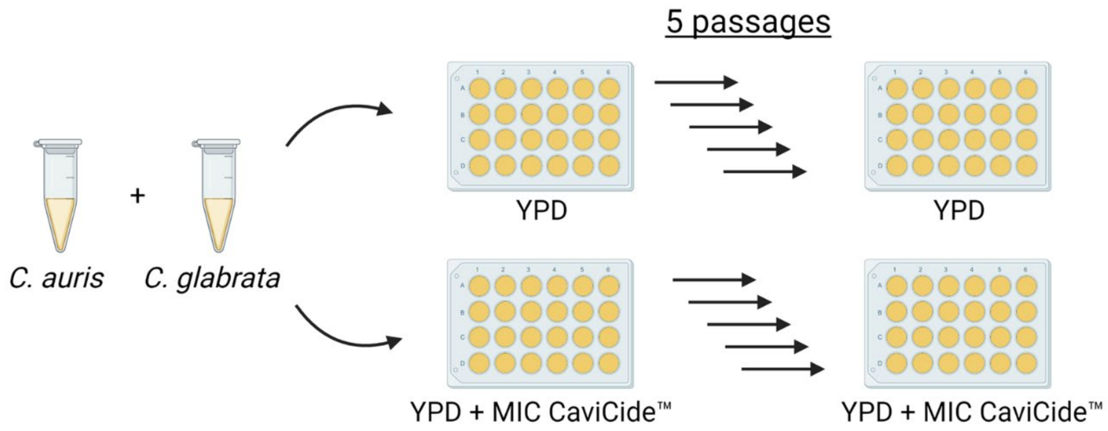

Figure S7. Passaging experimental setup with defined ratio of *C. auris* and *C. glabrata*. Created with BioRender.com.

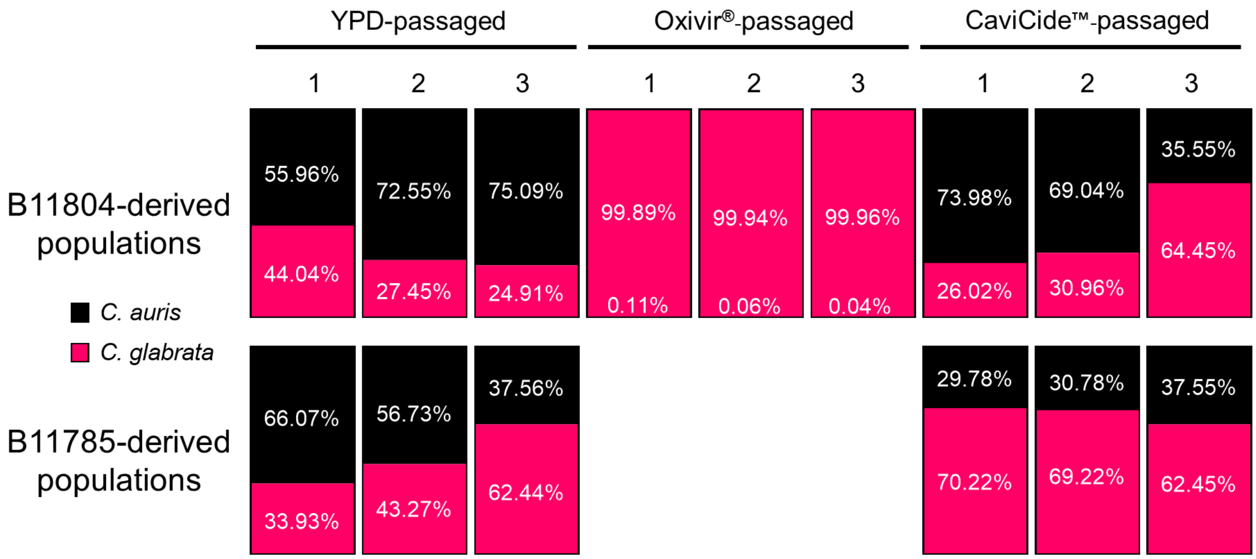

Figure S8. Retrospective analysis of populations from passaging experiment 2 with relative proportions of each species. Oxivir®-passaged, B11785-derived populations' glycerol stocks were no longer recoverable.
